# Seroprevalence of Hepatitis E virus in children and adolescents living in urban Bogotá: An explorative cross-sectional study

**DOI:** 10.3389/fpubh.2023.981172

**Published:** 2023-02-07

**Authors:** Nathalie Verónica Fernández Villalobos, Barbora Kessel, Johanna Carolina Torres Páez, Julia Strömpl, Tobias Kerrinnes, Fernando Pio de la Hoz Restrepo, Monika Strengert, Gérard Krause

**Affiliations:** ^1^Department of Epidemiology, PhD Programme, Helmholtz Centre for Infection Research (HZI), Braunschweig-Hannover, Hannover, Germany; ^2^Department of Epidemiology, Helmholtz Centre for Infection Research (HZI), Braunschweig, Germany; ^3^Department of RNA-Biology of Bacterial Infections, Helmholtz Institute for RNA-based Infection Research (HIRI), Würzburg, Germany; ^4^Departamento de Salud Pública, Facultad de Medicina, Universidad Nacional de Colombia, Bogotá, Colombia; ^5^Twincore, Centre for Experimental and Clinical Infection Research, A Joint Venture of the Hannover Medical School and Helmholtz Centre for Infection Research, Hannover, Germany; ^6^German Center for Infection Research (DZIF), partner site: Braunschweig-Hannover, Braunschweig, Germany

**Keywords:** adolescents, children, Colombia, *Paslahepevirus balayani* (previously Hepatitis E virus), seroprevalence, risk factors, surveillance, transmission

## Abstract

The majority of Hepatitis E Virus (HEV)-related studies are carried out in adults whereas information about HEV seroprevalence, clinical disease manifestation, molecular epidemiology, and transmission patterns in children is limited. To estimate HEV seroprevalence among scholar children living in an urban setting and to analyze risk factors for an infection, we invited children aged 5–18 years from Bogotá (Colombia) for a cross-sectional survey. We collected self-reported data on demographics, social, clinical, and exposure variables in a structured interview. Venous blood samples were analyzed with two commercially available ELISAs for HEV-specific IgG antibodies. Among the 263 participants, we found three HEV IgG-reactive samples (1.1%) using both assays. We additionally characterized the samples for HEV IgM using a commercially available IgM ELISA and for HEV RNA. Here, we found one IgM-reactive sample, which was also reactive for IgG. In contrast, none of the IgM- and IgG-reactive sera samples showed detectable RNA levels indicating HEV exposure had not been recently. All participants reported access to drinking water and sanitary systems in their households and frequent hand washing routines (76–88%). Eighty percent of children reported no direct contact with pigs, but occasional pork consumption was common (90%). In contrast to the majority of studies performed in Colombian adults, we found a low unadjusted HEV seroprevalence of 1.1% (95% CI: 0.3–3.6%) for both HEV IgG ELISAs in our study population. While the majority of participants reported pork consumption, we speculate in the absence of viral RNA for genotyping in the affected individuals, that existing access to drinking water and sanitary systems within our study group contribute to the low HEV seroprevalence.

## 1. Introduction

*Paslahepevirus balayani* (HEV), previously known as Hepatitis E virus, is responsible for a liver disease that affects ~ 20 million people worldwide, especially in low- and middle-income countries with poor socioeconomic conditions such as lack of sanitation, low-quality drinking water, or food supply (1, 2). While HEV liver disease is usually self-limiting with mild symptoms, it can result in severe acute hepatitis, extrahepatic disorders, chronic hepatitis leading to fibrosis/cirrhosis, and fulminant hepatitis in some individuals (3). In particular, pregnant women face an increased risk of fulminant hepatitis with a mortality rate of 26.9% (4), whereas 66% of immunocompromised solid-organ transplant recipients develop a chronic course of infection (5).

HEV as a single-stranded RNA virus can be grouped in 8 genotypes (HEV-1-HEV-8) within the *Hepeviridae* family, *Orthohepevirinae* subfamily, *Paslahepevirus* genus, and *balayani* specie (6), but only genotype 1–4 and 7 infect humans (2, 7). HEV-1 and HEV-2 are dominant in low-middle-income countries within Africa and Asia where they cause both sporadic cases and larger outbreaks (8). While the fecal-oral HEV-1 and HEV-2 transmission route by contaminated water has been ascertained, HEV-3 and HEV-4 infection appear to be primarily associated with the consumption of contaminated or undercooked meat, or direct contact with an infected animal such as pigs, deer, or wild boar (8). Additionally, HEV-3 transmission after blood transfusions has also been documented (8–10). HEV-7 infection has been predominantly reported in camels (11), but one study has also found a patient to be infected by HEV-7 after consuming camel meat and milk (7). Other studies have also reported serological and molecular evidence of human infections with members of the *Rocahepevirus ratti* species, especially with the genotype C1 (12–17).

Global HEV seroprevalence estimates strongly vary and range from 0.25% to 74.76% (8). Those discrepancies are not only attributable to differences in hygienic standards, access to sanitation, or in zoonotic exposures, but also dependent on which serological assay is used to determine a previous HEV exposure (8, 18, 19). Although infections are thought to occur mainly in late childhood or young adulthood (20, 21), there are few studies that actually investigate HEV disease burden in children (22), and information about HEV seroprevalence, clinical manifestations, molecular epidemiology and transmission patterns in this population are equally less well examined (22, 23).

To address this gap, we performed a HEV seroprevalence study in children from Bogotá, Colombia. Colombia is an upper-middle-income country with clear social determinants of health inequalities especially between the rural and urban regions (24, 25). Bogotá, the country's capital has the lowest Unsatisfied Basic Needs indicator and the highest percentage coverage of public services in Colombia (26). To date, a limited number of studies has examined HEV disease burden in Colombian adults. Molecular and/or serological evidence of HEV infection has been detected in human sera or feces samples from acute hepatitis patients from different Colombian cities (27, 28), blood donors from Antioquia (29), swine farm workers from Medellin (30), and in slaughtered pigs or pig feces in Antioquia (31) or Medellin (32), respectively. HEV genome was also detected in both waste and drinking water in Antioquia (33). Concerning genotype distribution in Colombia, HEV-3 was first characterized in a 2008–2009 study (27, 34). HEV seroprevalence estimates were variable, while two studies in viral hepatitis patients showed anti-HEV proportions of 7.5% and 25.3% for IgG and of 1.74% and 5.6% for IgM (27, 33), the reported HEV IgG seroprevalence was 45.2% in blood donors (29).

We provide for the first time data on HEV seroprevalence estimates among scholar children living in the urban setting of Bogotá, and analyzed risk factors for infection by association with demographics, social, clinical, and exposure variables. Data about HEV seroprevalence in children is essential to understand the variable levels of seroprevalence not only observed in Colombian adults, but it also provides information for health authorities on the current extent of HEV infections to support the possible inclusion of HEV in the diagnosis and management plan of viral hepatitis in Colombia and to create control and prevention strategies for fecal-oral and zoonotic transmission.

## 2. Materials and methods

### 2.1. Study population

We designed a cross-sectional study, which was carried out in cooperation with the Universidad Nacional de Colombia (UNAL) from February 2020 until March 2021 in Bogotá, Colombia. Based on the division of Bogotá in 20 localities and 6 stratum areas (35, 36), we designed a two-stage cluster random sampling considering localities (37), and schools (38). The software Epidat version 4.2 was used to calculate a sample size of 280 (39) based on an estimate of 1.5 million individuals living in Bogotá aged between 5 and 18 years, an expected HEV seroprevalence of 3%, with a margin error of 2%, and confidence level of 95% (40).

Children and adolescents (further referred to as children) from 5 to 18 years old were invited to participate by advertising the study in different schools through electronic and paper-based documents. Prior to initiating the study, participating parents or legal guardians provided written informed consent. Inclusion criteria were to live in Bogotá, to study at the selected schools, and to have the authorization and company of a parent or legal guardian. We excluded children with any predisposition for bleeding, blood clots, cognitive deficits that prevent giving informed assent or consent, or suffering from primary or secondary immunodeficiency. Based on the socioeconomics characteristics of the respective population, we considered localities in Bogotá that had all strata represented (Suba, Usaquén, and Chapinero), and those with low socioeconomic conditions (San Cristóbal, Ciudad Bolívar, Usme, Bosa, and Santa Fe) for taking part in the study (41). Within the pre-identified localities, we randomly pre-selected two localities Ciudad Bolívar and Usaquén and different schools within those areas. However, due to the COVID-19 pandemic and the ensuing low numbers of participants, we invited other localities and schools to take part in the study (Supplementary Table 1). The COVID-19 pandemic also led to the exclusion of children and companions with risk factors or comorbidities such as diabetes, hypertension and others (Supplementary Table 2), or acute respiratory symptoms.

The study was conducted in line with the Declaration of Helsinki, and followed STROBE guidelines (42). It was approved by the Comité De Ética De Investigacion De La Facultad De Medicina, Universidad Nacional de Colombia, Bogotá, Colombia (N°.009-125-19) and by the Ethics Committee of Hannover Medical School, Hannover, Germany (Nr.9254_BO_K_2020).

### 2.2. Data collection and management

We collected self-reported data on demographic, social, clinical, and exposure variables through a structured questionnaire; using REDCap 7.3.6 electronic data capture tools hosted at Unidad de Informática y Comunicaciones - Facultad de Medicina - Universidad Nacional de Colombia (43, 44). Details are listed in Table 1.

**Table 1:** General characteristics of the participants.

**Characteristics of the participants**		**263 (100%) All participants *n* (%)**	**260 (98.9%) HEV IgG non-reactive participants *n* (%)**	**3 (1.1%) HEV IgG reactive participants *n* (%)**
**Social and demographic characteristics**				
Age in years: Median (IQR)		9 (8-11)	9 (8-11)	8 (7-9)
Sex	Male	142 (54.0)	141 (54.2)	1 (33.0)
	Female	121 (46.0)	119 (45.8)	2 (67.0)
School type	Public	234 (89.0)	231 (88.8)	3 (100.0)
	Private	29 (11.0)	29 (11.2)	0 (0.0)
Social security affiliation	Subsidized	38 (14.5)	38 (14.6)	0 (0.0)
	Contributory	217 (82.5)	214 (82.3)	3 (100.0)
	Unaffiliated	8 (3.0)	8 (3.1)	0 (0.0)
Socioeconomical strata^†^	One	4 (1.5)	4 (1.5)	0 (0.0)
	Two	93 (35.4)	91 (35.0)	2 (67.0)
	Three	163 (61.9)	162 (62.3)	1 (33.0)
	Four	2 (0.8)	2 (0.8)	0 (0.0)
	Unknown	1 (0.4)	1 (0.4)	0 (0.0)
Income^‡^	Between one and two minimum wages	174 (66.2)	171 (65.9)	3 (100.0)
	2–6 minimum wages	69 (26.2)	69 (26.5)	0 (0.0)
	More than 6 minimum wages	5 (1.9)	5 (1.9)	0 (0.0)
	Do not want to inform	11 (4.2)	11 (4.2)	0 (0.0)
	Unknown	4 (1.5)	4 (1.5)	0 (0.0)
Country of birth	Colombia	251 (95.4)	248 (95.4)	3 (100.0)
	Venezuela	10 (3.8)	10 (3.8)	0 (0.0)
	Other	2 (0.8)	2 (0.8)	0 (0.0)
**Behavioral characteristics**				
Mother occupation	Occupation with animal/soil contact	3 (1.1)	3 (1.1)	0 (0.0)
	Other	259 (98.5)	256 (98.5)	3 (100.0)
	Unknown	1 (0.04)	1 (0.4)	0 (0.0)
Father occupation	Occupation with animal/soil contact	2 (0.8)	2 (0.8)	0 (0.0)
	Other	229 (87.1)	226 (86.9)	3 (100.0)
	Unknown	32 (12.1)	32 (12.3)	0 (0.0)
Contact with pigs	Yes	52 (19.8)	52 (20.0)	0 (0.0)
	No	209 (79.4)	206 (79.2)	3 (100.0)
	Unknown	2 (0.8)	2 (0.8)	0 (0.0)
Pork consumption	Never	12 (4.6)	11 (4.2)	1 (33.0)
	Occasionally	236 (89.7)	234 (90.0)	2 (67.0)
	Usually	14 (5.3)	14 (5.4)	0 (0.0)
	Always	1 (0.4)	1 (0.4)	0 (0.0)
Drinkable water	Bottled	42 (16.0)	41 (15.8)	1 (33.3)
	Filtered	30 (11.4)	29 (11.2)	1 (33.3)
	Boiled	71 (27.0)	70 (26.9)	1 (33.3)
	Tap water	120 (45.6)	120 (46.1)	0 (0.0)
Hand washing after the toilet	Occasionally	27 (10.3)	27 (10.4)	0 (0.0)
	Usually	74 (28.1)	72 (27.7)	2 (67.0)
	Always	160 (60.8)	159 (61.1)	1 (33.0)
	Unknown	2 (0.8)	2 (0.8)	0 (0.0)
Hand washing before eating	Never	5 (1.9)	5 (1.9)	0 (0.0)
	Occasionally	55 (20.9)	53 (20.4)	2 (67.0)
	Usually	90 (34.2)	90 (34.6)	0 (0.0)
	Always	112 (42.6)	111 (42.7)	1 (33.0)
	Unknown	1 (0.4)	1 (0.4)	0 (0.0)
Recreational swimming in rivers or streams	Yes	155 (58.9)	154 (59.2)	1 (33.0)
	No	108 (41.1)	106 (40.8)	2 (67.0)
**Health-related characteristics**				
Blood transfusion	Yes	1 (0.4)	1 (0.4)	0 (0.0)
	No	262 (99.6)	259 (99.6)	3 (100.0)
Jaundice	Yes	2 (0.8)	2 (0.8)	0 (0.0)
	No	261 (99.2)	258 (99.2)	3 (100.0)
Viral hepatitis diagnosis	Yes	2 (0.8)	2 (0.8)	0 (0.0)
	No	261 (99.2)	258 (99.2)	3 (100.0)
Hepatitis symptoms	Yes	16 (6.1)	16 (6.2)	0 (0.0)
	No	247 (93.9)	244 (93.8)	3 (100.0)

^†^One represents the lowest strata and six the highest.

^‡^Colombian minimum wage ~ 280 USD.

In addition, we took ~ 5 ml of venous blood by venipuncture using an S-Monovette (cat no: 03.1397, Sarstedt, Sarstedt, Germany). Prior to freezing at <-20°C, samples were allowed to clot for 30 min at ambient temperature, and then centrifuged at 2,000 g for 10 min to obtain serum. In the absence of a gold standard for identification of anti-HEV IgG antibodies, two commercially available ELISAs were selected:

1. HEV IgG ELISA [cat no: 88 03 30, Axiom Diagnostic, Bürstadt, Germany; developed by Wantai, Beijing, China (19, 45)]: with a reported sensitivity of 93% (calculated with 90/91 patients with confirmed HEV infection) and a specificity of 99% (calculated with 414/418 blood donor samples) determined by Norder et al. (46).
2. recomWell HEV IgG [cat no: 5004, Mikrogen Diagnostik, Neuried, Germany]: with a reported sensitivity of 98.9% (calculated with 88/89 patients with acute HEV infections) and a specificity of 98.5% (calculated with 132/134 blood donor sera samples) defined by the manufacturer (47).

While the recomWell HEV IgG ELISA uses recombinant peptides of HEV ORF2/ORF3 genotypes 1 and 3 (46, 47) as antigens, the Axiom HEV IgG ELISA limits itself to the carboxy-terminal region of the ORF2 genotype 1 Burmese strain (46). Samples were measured once and classified as reactive for anti-HEV IgG based on an assay-specific signal to cut-off (S/CO) value of above 1.1 or 1.2 for the Axiom and the Mikrogen ELISA, respectively. HEV IgG-reactive samples after the first screening of all samples were re-measured in triplicates to confirm reactivity.

Because of the unavailability of the World Health Organization Reference Reagent for Hepatitis E Virus Antibody (NIBSC code: 95/584), we included two other available NIBSC quality control reagents QCRTHAVQC1 - Total Anti-Hepatitis A Virus Quality Control Reagent Sample 1 (NIBSC code: 17/B725) and QCRHEVQC1 - Anti-Hepatitis E Quality Control (NIBSC code: 17/B723) on every ELISA plate to monitor assay performance (Supplementary Table 3).

Next to an analysis for HEV IgG, all samples were also analyzed for HEV IgM using the recomWell HEV IgM [cat no: 5005, Mikrogen Diagnostik, Neuried, Germany, with a reported sensitivity of 98.9% (calculated with 87/89 patients with acute HEV infection) and a specificity of 98.5% (calculated with 354/359 of patients with a suspected non-HEV infection and blood donors) (47)], as IgM reactivity indicates a more recent infection (48). All IgM-reactive/-borderline samples in the first measurement were re-measured twice for an unequivocal IgM result. All IgG- and IgM-reactive samples were then further analyzed with the IVD-certified RealStar HEV RT-PCR Kit 2.0 (cat no: 272013, Altona Diagnostics, Hamburg, Germany) for the detection and, if applicable, the quantification of HEV-specific RNA. Prior to qRT-PCR analysis, nucleic acid from sera samples was isolated using the QIAamp MinElute Virus Spin Kit (cat no: 57704, Qiagen, Hildesheim, Germany). All laboratory analysis were performed according to the manufacturer's instruction. A more detailed description of the procedures can be found in Supplementary material.

### 2.3. Statistical analysis

Presence or absence of HEV IgG was defined as the main study outcome and considered as dependent variable. As exposures, we studied different socioeconomical and behavioral aspects, with special emphasis in fecal-oral and zoonotic transmission. We summarized categorical variables as counts and percentages, and continuous variables as medians and inter-quartile ranges (IQR). Due to only three reactive samples, we refrained from carrying out formal statistical tests assessing the association between HEV IgG reactivity and socioeconomical and behavioral variables.

We calculated the proportion of HEV IgG-reactive samples with their 95% confidence intervals (95% CI), score method with Yates' correction, function prop.test() in the R package “stats” (49) for each ELISA. After this, the crude seroprevalence was adjusted for the respective test's sensitivity and specificity as proposed by Lang and Reiczigel (50) using the R package “asht” (51). For calculation of those adjusted seroprevalence estimates, we used the sensitivity and specificity values determined by Norder et al. (46) for the Axiom assay and for the recomWell assay those provided by the manufacturer (47), as the latter reports information from an updated recomWell assay with altered performance characteristic that was also utilized in our study. We also assessed the inter-rater reliability between the two HEV IgG ELISAs by calculating Fleiss's *k* (52) with the R package “irrCAC” (53).

All statistical analysis were performed using RStudio (54) version 4.0.2, and the geographical representation was done using ArcGIS version 10.8.1 using the boundaries provided by Humanitarian Data Exchange (55) and data from Datos Abiertos Bogotá (56). HEV IgG/IgM S/CO calculation and quantification of HEV RNA were performed in Excel 2016 (Microsoft, Redmond, USA) or in GraphPad Prism 9.4.1 (GraphPadInc, SanDiego, USA), respectively.

## 3. Results

**Figure 1:**
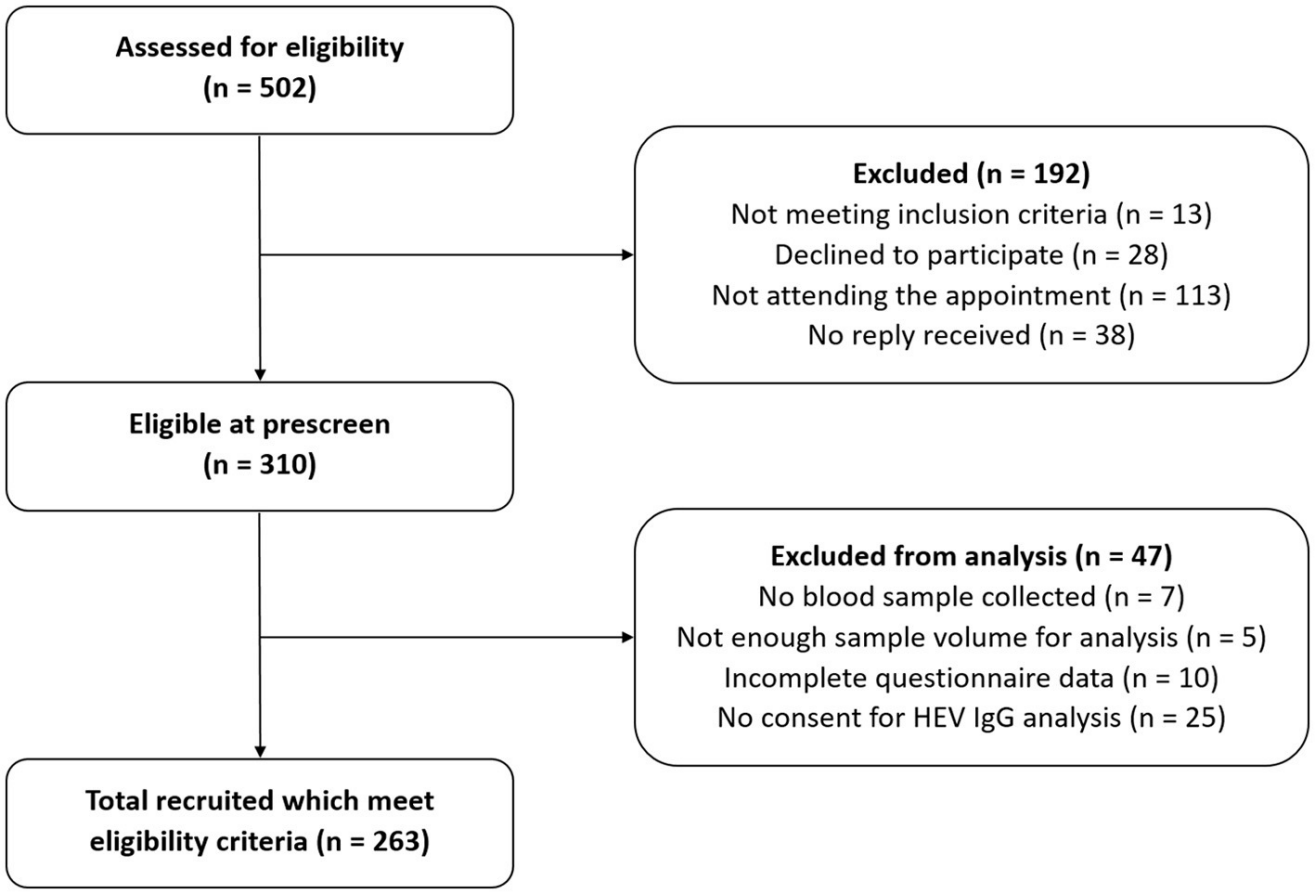
Flow chart of participants' selection.

During the recruitment phase, 502 people showed interest to participate in the study. After the first contact with the team, 192 (38%) people were excluded because they did not meet the inclusion criteria, declined participation, did not attend the appointment, or did not reply further. Among the remaining 310 people (62%), 263 of them (85%) were included into the study, as they had a sufficiently complete data set suitable for analysis (Figure 1). The median age of those participants was 9 years (interquartile range 8–11 years), and 142 (54.0%) participants were male. Other characteristics of the participants are presented in Table 1.

We detected three HEV IgG-reactive samples with the recomWell HEV IgG ELISA and the Axiom HEV IgG ELISA in our final study population resulting in a crude seroprevalence of 1.1% (95% CI: 0.3–3.6%). When adjusting this crude estimate by each assay sensitivity and specificity, we found a seroprevalence of 0.0% (95% CI: 0.0–2.6%) for the recomWell assay and of 0.2% (95% CI: 0.0–2.6%) for the Axiom assay. When analyzing the concordance between serological test results (Table 2), we obtained a Fleiss's *k* agreement coefficient of 1, which demonstrates a perfect agreement between the assays.

**Table 2:** Concordance of test results between both HEV IgG ELISAs.

**Axiom assay results**	**RecomWell assay results**		
	**Positive**	**Negative**	**Total (min–max S/CO)**
Positive	3	0	3 (9.36–14.54)
Negative	0	260	260 (−0.01–0.89)
Total (min–max S/CO)	3 (4.51–8.55)	260 (0.01–0.87)	263

**Figure 2:**
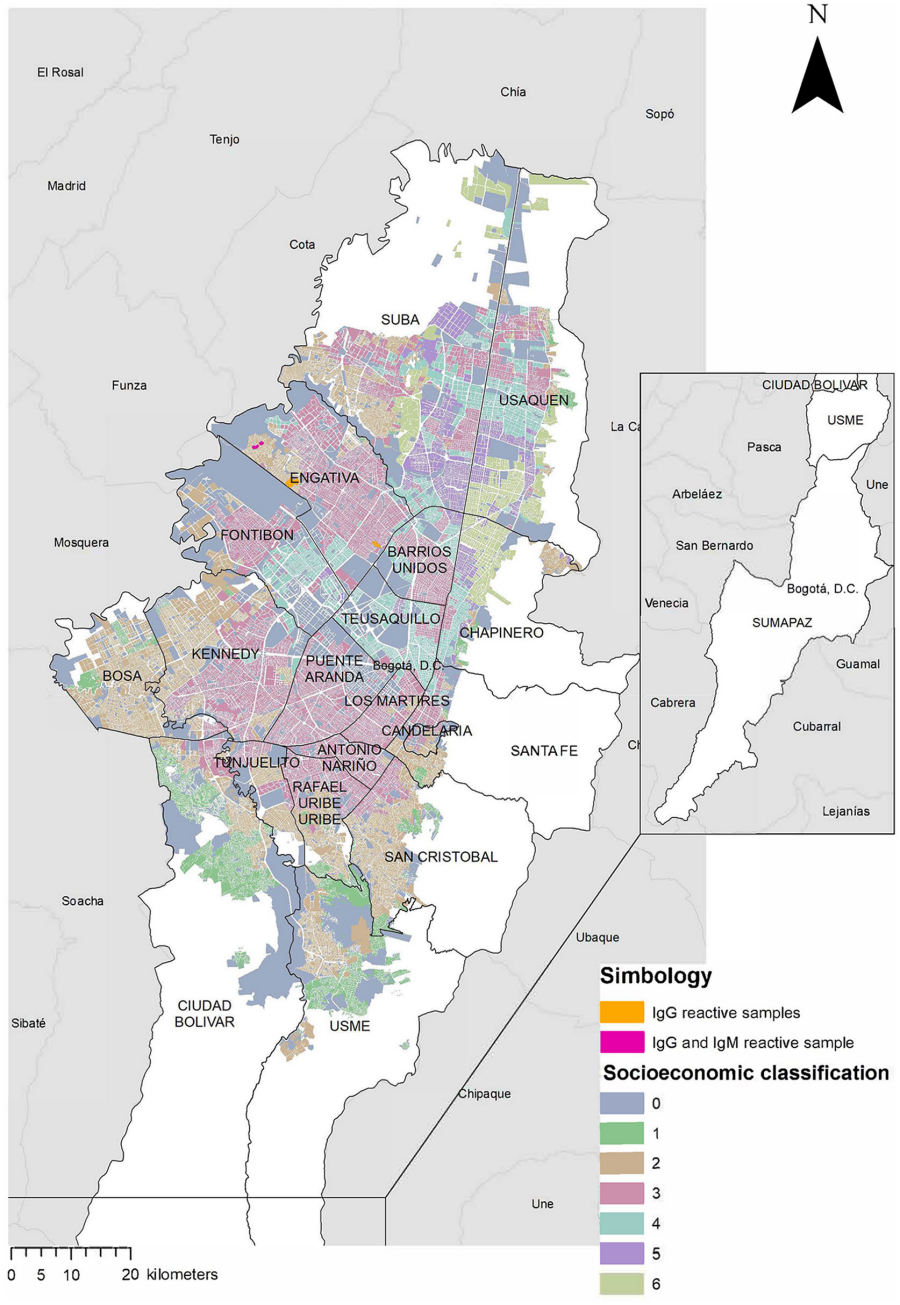
Neighborhoods in Bogotá with HEV antibody-reactive samples. The map shows the distribution of the localities and their socioeconomic classification. Neighborhoods with HEV IgG-reactive samples are highlighted in orange, the neighborhood with a HEV IgG- and IgM-reactive sample is highlighted in fuchsia.

All HEV IgG-reactive samples originated from participants born and raised in Colombia, who lived in areas of socioeconomic strata 2 and 3 (Table 1). Their parents received an income between one and two minimum wages (280–560 USD). All HEV IgG-reactive samples were from the locality Engativá but from different neighborhoods (Figure 2). None of the individuals with HEV IgG-reactive samples reported having an earlier blood transfusion, having suffered previously from jaundice or any other hepatitis-related symptoms, or having a diagnostic hepatitis panel done before our study took place.

All HEV IgG-reactive participants reported access to good-quality drinking water and sanitary systems in their homes making handwashing before eating food and after using the toilette possible. Pork consumption was reported by two people (67%), while recreational swimming in rivers or lakes was reported by one person (33%) (Table 3). Due to the low number of reactive cases, we refrained from further statistical evaluation to examine if age, gender, or other examined behavioral or environmental factors such as pork consumption, were associated with an earlier HEV infection.

**Table 3:** Characteristics of the HEV IgM- and IgG-reactive samples within the study population.

**Results**	**Sex**	**Age group**	**Contact with pigs**	**Pork consumption**	**Most frequent source of drinking water**	**Hand washing after the toilet**	**Hand washing before eating**	**Recreational swimming in rivers or streams**	**Mean IgG Axiom S/CO**	**Mean IgG Mikrogen S/CO**	**IgM Mikrogen S/CO**
IgG^+^	Female	5–8	No	Never	Bottled	Usually	Occasionally	No	9.70	4.51	0.38
IgG^+^	Male	5–8	No	Occasioally	Boiled	Always	Always	No	9.36	5.35	0.25
IgG^+^ & IgM^+^	Female	9–11	No	Occasionally	Filtered	Usually	Occasionally	Yes	14.54	8.55	1.43

After performing the IgM analysis, we found one reactive sample resulting in crude seroprevalence of 0.4% (95% CI: 0.0–2.4%) and an adjusted seroprevalence of 0% (95% CI: 0.0–1.3%). This reactive IgM sample was also reactive for IgG (Table 3). When analyzing those sera for HEV RNA by quantitative RT-PCR, no amplification traces were detectable in any of the samples.

## 4. Discussion

We explored the HEV seroprevalence in children aged 5–18 years living in an urban setting of Bogotá, Colombia together with social, clinical, and exposure variables to analyze risk factors for a HEV infection. We found an unadjusted HEV seroprevalence of 1.1% (95% CI: 0.3–3.6%) in our study population using two HEV IgG ELISAs. Our low HEV seroprevalence is in line with the few other studies performed in children in Latin America (57). While the first of two studies conducted in Argentina found a crude HEV IgG seroprevalence of 0.15% in participants from urban Buenos Aires with a mean age of 6.4 (58), the second one found a crude seroprevalence of 1.7% in participants from the rural Chaco Province with a median age of 14 years (59). HEV crude seroprevalence in children from urban Santa Cruz, Bolivia was 1.7% (95% CI: 1.5–1.8%) (60), in which all HEV IgG-reactive participants were exclusively from the poorest social class and between 13 and 18 years old. Other studies have shown higher seroprevalences. A study from Mato Grosso State, Brazil where a series number of hepatitis cases occurred in 1997 and 1998, reported a HEV IgG seroprevalence of 4.5% (95% CI 2.9–6.9%) in children aged 2–9 years (61), while a study from Mexico with representative regional and socioeconomic sampling found a HEV IgG seroprevalence of 4.4% in 5–14 year old children with increased Odds Ratios for age, type of community, and educational level (62). Even though our low seroprevalence is in line with similar studies in other Latin American countries, our study design did not allow us to identify an increase of seroprevalence with age, as shown by the study in Mexico (62). The latter study selected serum samples from a National Serologic Survey performed in 1987 and 1988, which included 3,549 participants aged 1 to 29 years old (62). Evidence suggests that increasing age is a risk factor for HEV infection (57). A systematic review by Belei et al. found that HEV seroprevalence estimates in individuals between 15 and 30 years can reach about 30% (63). Kmush et al. studied HEV seroprevalence in both children and adults and found an overall seroprevalence of HEV antibodies among adults of 9.52% (95% CI: 3.58–19.59%) in contrast to a seroprevalence of 0.7% (95% CI: 0.15–2.09%) in children (64).

As observed by Kmush et al. in New York, United States, our low HEV seroprevalence in children contrasts to increased levels of HEV seropositivity in the adult population of Colombia where study reported a seroprevalence of 1.74% for IgM antibodies and of 7.5% for IgG in 344 human sera samples from 16 Colombian departments (28). Another study that included 1,097 sera samples from 32 departments from patients with active viral hepatitis described a seroprevalence of 31.2% for IgG and of 11.5% for IgM (27). A further study performed in Medellin, Antioquia found nine (22.5%) cases of HEV infection in 40 fecal samples of patients with a clinical diagnosis of viral hepatitis using nested RT-PCR (65). In contrast to the previous studies, one study performed in 42 blood donors from the Municipality of Yarumal, Antioquia has identified 19 (45.2%) HEV IgG-reactive sera samples, but none for IgM (29). It is noteworthy that the majority of studies examining HEV epidemiology in Colombia not only in humans, but also in swine or in water samples originate from Antioquia. Interestingly, this region is the department with the highest pork production activity of 43.4% in Colombia (66). For environmental samples, one study in Antioquia detected HEV genome in 23.3% (7/30) of the samples from drinking water plants and in 16.7% (5/30) from sewage by RT-PCR (33). Several further studies provide evidence for HEV presence in pigs or pig products in Antioquia, one study reported that 41.3% of pig livers from slaughterhouses and 25% of livers from grocery stores in Medellin tested positive for HEV RNA by RT-PCR (67). Another study performed in fecal samples from 210 animals from 30 pig farms of Antioquia found that 100% of the samples were reactive for IgG antibodies, and 57% for IgM antibodies. Evidence of HEV genome was found in 26% of pig feces (31). A last study that included blood samples from pigs of Antioquia found 100% seropositivity for IgG antibodies and 82.06% for IgM antibodies using a commercial ELISA kit (32).

The most recent systematic reviews and meta-analysis' have identified risk factors for a HEV infection as consummation of raw meat, exposition to soil, having had a blood transfusion, travel to endemic areas, contact with dogs, living in rural areas and receiving lower level of education on a global level (8) and focused on the Americas as increasing age, contact with pigs and meat products, and low socioeconomic conditions (57). Our discrepant levels of HEV seropositivity in Colombian children and adults are reflected in the above identified risk factors where potential occupation and/or living in (rural) areas with pig farming and meat production are given. However, other factors such as improved hygiene standards, access to sanitation, or changes in behavioral conducts such as increased awareness of risks from undercooked meat combined with avoiding consumption of raw meat in adults/parents might contribute also to the low seroprevalence in the current children population. Longitudinal follow-up exams in regular intervals in our study population until adulthood could contribute to further clarify those discrepant levels of HEV seroprevalence. As already shown in other studies (68), none of HEV-IgG reactive participants of the current study reported any hepatitis-related symptoms indicating that HEV infection is mostly asymptomatic in children. Interestingly, the three participants with detectable HEV IgG in both assays showed high S/CO values pointing toward a robust immune response. Unfortunately, we were not able to convert our semi-quantitative antibody titers for further standardization using the World Health Organization Reference Reagent for Hepatitis E Virus Antibody due to its current unavailability (69) to substantiate our observation. Interestingly, we observed no differences in the number of HEV IgG-reactive samples between the two immunoassay used which differ in the peptide antigens for antibody capture. This is in contrast to an observation of Pezzoni et al. who found that 12% of tested swine sera were only reactive toward ORF3 protein (70). In addition, we observed slight discrepancies in adjusted seroprevalences in the two commercially developed anti-HEV IgG immunoassays. Those originated from different sensitivity and specificity, which can even be observed when the same sample sets were used for validation (19, 46). Those discrepancies in assay performance might have further implications in particular in a low-prevalence setting as ours and underline the need for standardization in HEV serology. Adjusting seroprevalence estimates for an assay's sensitivity and specificity, as done by us, does not only reflect better the underlying population seroprevalence, but it also makes results across studies directly comparable.

We found one HEV IgM-reactive sample, and no HEV-specific RNA was detectable in any of the IgM- or IgG-reactive sera samples. The only IgM-reactive sample was also reactive for IgG, which might represent an acute infection case, even in the absence of detectable RNA levels. While IgM antibodies decline more rapidly after an acute infection and can be detectable only for a few months after onset of symptoms, IgG antibodies can persists for at least 1 year (71, 72). RNA on the contrary declines even more rapidly, and it is not detectable in the serum by day 20 after onset of symptoms (73). Therefore, the other two HEV IgG-reactive samples might indicate an even earlier infection as no IgM were identified in them.

Our study has several limitations. Although our sample size is comparable to the majority of other studies in the region with a population screened between 99 and 1,848 children (22, 74), our recruitment and sampling processes were hampered by massive and long-lasting school closure due to the COVID-19 pandemic. We had to include more localities and schools than those previously selected by changing our random to a convenient sampling. Nevertheless, we were able to gather diverse samples in terms of sociodemographic characteristics such as different strata, broad age ranges, and localities of origin. Moreover, our study included self-reported medical and behavioral information, which may be inaccurate and threatened by self-reporting bias (75). We could not find any factor associated with HEV reactivity due to the few observations within groups in our study. Lastly, we only examine B-cell and not T-cell mediated immune responses, which also offer protection from reinfection (76). While the presence of antibodies is regarded as immune correlates of protection against HEV infection, we equally cannot provide insight into their persistence due to the lack of a longitudinal follow-up component in our study design. We were also not able to define the HEV genotype responsible for the infection to gain further insight into mode of transmission. Data on viral circulation in non-human samples were neither gathered nor available to correlate with findings in human bio-samples.

In conclusion, the unadjusted HEV IgG seroprevalence in the study population was 1.1% (95% CI: 0.3–3.6%) using both assays; the recomWell HEV IgG ELISA which detects antibodies reactive to ORF2 and ORF3 protein and the Axiom assay which only uses the ORF2 peptide as antigen. We can highlight that the participants living in an urban setting of Bogotá, Colombia, have good access to drinkable water and sanitary systems, have good hand-washing practices, rare contact with pigs, and moderate consumption of pork. As serological testing cannot define the viral genotype responsible for the previous infection, we speculate that those factors might explain the low HEV infection numbers found in our study.

## Data availability statement

The raw data supporting the conclusions of this article have been provided by the authors in a public repository (https://zenodo.org/badge/latestdoi/559831870).

## Ethics statement

The study involving human participants was reviewed and approved by the Comité De Ética De Investigacion De La Facultad De Medicina, Universidad Nacional de Colombia, Bogotá, Colombia (N°.009-125-19) and the Ethics Committee of Hannover Medical School, Hannover, Germany (Nr.9254_BO_K_2020). Written informed consent to participate in this study was provided by the participants' legal guardian/next of kin.

## Author contributions

GK, FH, TK, NF, MS, and JT designed the study. NF and JT executed the study and collected data. GK, MS, and FH supervised the study. MS and JS performed the laboratory experiments. MS supervised and coordinated laboratory work and analyzed laboratory data. NF cleaned the database and prepared data for analysis. BK, MS, and NF analyzed and interpreted the data. NF and MS prepared the initial manuscript. MS and BK verified the underlying data and provided advice on data analysis. All authors have revised the manuscript, read, and approved the final version. All authors confirm full access to all the data in the study and accept responsibility to submit for publication.
