# Supplementary material for: Seroprevalence of Hepatitis E virus in children and adolescents living in urban Bogotá: An explorative cross-sectional study
Source: Front Public Health. 2023 Feb 7;11:981172. doi: 10.3389/fpubh.2023.981172 (PMC9943700; doi:10.3389/fpubh.2023.981172)
Supplement: Supplementary file 1 [file Data_Sheet_1.pdf]

Supplementary Material - Seroprevalence of Hepatitis E virus in children and adolescents living in urban Bogotá: an explorative cross-sectional study

Nathalie Verónica Fernández Villalobos[1], Barbora Kessel[2], Johanna Carolina Torres Páez[1], Julia Strömpl[2], Tobias Kerrinnes[3], Fernando Pio de la Hoz Restrepo[4], Monika Strengert[2,5,*,†], Gérard Krause[2,5,6,†]

[1] Department of Epidemiology, PhD Programme, Helmholtz Centre for Infection Research (HZI), Braunschweig-Hannover, Germany
[2] Department of Epidemiology, Helmholtz Centre for Infection Research (HZI), Braunschweig, Germany
[3] Department of RNA-Biology of Bacterial Infections, Helmholtz Institute for RNA-based Infection Research (HIRI), Würzburg, Germany
[4] Universidad Nacional de Colombia, Facultad de Medicina, Departamento de Salud Pública, Bogotá, Colombia
[5] Twincore, Centre for Experimental and Clinical Infection Research, A Joint Venture of the Hannover Medical School and Helmholtz Centre for Infection Research, Hannover, Germany
[6] German Center for Infection Research (DZIF), partner site: Braunschweig-Hannover, Germany

[†] These authors share last authorship

*** Correspondence:**
Monika.Strengert@helmholtz-hzi.de

Supplementary participant's information

<u>Definition of socioeconomic strata in Colombia</u>

The Colombian government has regulated public services such as piped water, sewerage, sanitation, electricity, fuel gas distribution, basic public fixed telephony and local mobile telephony in the rural sector based on the residential properties (1). According to the § 102 of Law 142, "residential properties to which public services are provided shall be classified in a maximum of six socioeconomic strata as follows: 1) low-low, 2) low, 3) medium-low, 4) medium, 5) medium-high, and 6) high"[1]. This classification depends on the physical characteristics of the house and its immediate surrounding and urban context.

Supplementary Table 1. Localities of included schools and their socioeconomic strata

**Locality**	**Socioeconomic strata of the school**
Bosa	2
Ciudad Bolívar	3
Kennedy	3
Usaquén	3
Engativá	3

Supplementary Table 2. Exclusion factors from the study for both children and their companions from October 2020

**Comorbidities or conditions**		
Pregnancy	Other pulmonary diseases	Obesity
Companions above the age of 60	Neurologic diseases	Malnutrition
Hypertension	Severe Liver disease	Thyroid disease
Untreated asthma	Severe Kidney disease	Diabetes

Supplementary method and data section

Serological assays

HEV IgG ELISA (Axiom): All samples were analyzed according to the manufacturer's protocol using an identical assay lot. Briefly, samples, negative and positive controls were diluted 1:101 in specimen diluent on the provided microwell plate and incubated for 30 min at 37 °C. Negative and positive control samples provided by the manufacturer were run in triplicate or duplicate wells on each plate, respectively. After 5 manual wash cycles with 350 µl diluted wash buffer and a soaking time of 30 sec per cycle, 100 µl HRP-conjugate was added for an additional 30 min incubation step at 37 °C. Afterwards, before the addition of 50 µl of chromogen A and chromogen B solution per well, 5 manual wash cycles were performed as before. After a 15 min incubation step, 50 µl stop solution was added. Plates were read immediately on an infinite M200 Pro plate reader (Tecan, Männedorf, Switzerland) at an absorbance wavelength of 450 nm and a reference of wavelength of 630 nm (standard setting: 25 flashes). All 37 °C incubation steps were carried out in a temperature-controlled Heraeus incubator (Thermo Fisher Scientific, Waltham, USA).
Samples were classified as IgG-reactive, if their S/CO was >1.1 (630 nm- and blank-corrected OD value of sample / 630 nm and blank-corrected mean OD value of negative control + 0.16). HEV IgG-reactive samples after the first screening were re-measured in triplicates to confirm reactivity. All plates had to pass the below quality control criteria defined by the manufacturer to be considered a valid run:

- The OD value of the blank well, which contains only chromogens and stop solution is < 0.080 at 450 nm
- The OD value of the positive control must be ≥ 0.800 at 450/630 nm
- The OD value of the negative control must be < 0.100 at 450/630 nm

recomWell HEV IgG and IgM ELISA (Mikrogen): All samples were analyzed according to the manufacturer's protocol using an identical assay lot. Briefly, samples, IgG or IgM negative, IgG or IgM positive and the IgG or IgM cut-off control were diluted 1:101 in dilution buffer. 100 µl of those dilutions were added to the provided recomWell HEV IgG or IgM 12x8 wells micro plates and incubated for 1 h at 37 °C. IgG or IgM negative, IgG or IgM positive and the IgG or IgM cut-off control provided by the manufacturer were run in duplicate wells on each plate, respectively. After sample and control incubation, plates were washed 4 times with 300 µl diluted wash buffer with an HydroFlex plate washer (Tecan) before 100 µl of a 1:101 dilution of anti-human IgM or IgG-conjugate prepared in dilution buffer was added per well. After a 30 min incubation step at 37 °C and 4 additional wash cycles, 100 µl of TMP substrate was added per well. Substrate incubation for 30 min was performed at ambient temperature. After the addition of 100 µl stop solution, plates were read immediately on an infinite M200 Pro plate reader (Tecan) at an absorbance wavelength of 450 nm and a reference of wavelength of 650 nm (standard setting: 25 flashes). All 37 °C incubation steps were carried out in a temperature-controlled Heraeus incubator (Thermo Fisher Scientific).

Samples were classified as IgG- or IgM-reactive, if their S/CO was >1.2 (650 nm-corrected OD value of sample / 650 nm-corrected mean OD value of cut-off control sample). Samples with a S/CO between 1 and 1.2 were considered borderline. HEV IgG-reactive samples after the first screening of all samples were re-measured in triplicates to confirm reactivity. IgM-reactive and -borderline samples after the first screening were re-measured twice for an unequivocal results.
All plates had to pass the below quality control criteria defined by the manufacturer to be considered a valid run:

- The single OD values of the duplicate cut-off control were not allowed to deviate by more than 20 % from their average
- OD value - negative control ≤ 0.150
- Cut-off control OD value - negative control OD value ≥ 0.050
- Positive control OD value - cut-off control OD value ≥ 0.300

Molecular analysis of sera samples

Nucleic acid isolation: Nucleic acid from sera samples was isolated using the QIAamp MinElute Virus Spin Kit (cat no: 57704, Qiagen, Hildesheim, Germany) according to the manufacturer's instructions. Briefly, 150 µl of sera sample was used for isolation. For lysis, we used 200 µl AL-buffer with 28 µg/ml carrier RNA (cRNA) per sample. AL-buffer sample-lysates supplemented with 25 µl Qiagen Protease were pulse-vortexed for 15 sec and then incubated for 15 min at 56 °C in an Eppendorf Thermomixer Comfort (Eppendorf, Hamburg, Germany). Next, 250 µl of molecular-grade ethanol (cat no: 109301, SERVA Electrophoresis, Heidelberg, Germany) was added and the tubes were pulse-vortexed for 15 sec. The AL-buffer-sample-ethanol mixture was then incubated at ambient temperature for 5 min before being transferred into a QIAamp MinElute column, which was centrifuged for 1 min at 6000 x g in a Thermo Scientific Heraeus Tresco 17 centrifuge. After the centrifugation, the QIAamp Min Elute column was then washed by addition of 500 µl of AW1- or AW2-buffer or molecular-grade ethanol, respectively. Between each wash step, a centrifugation step (6000 x g, 1 min) was performed, the flow-through was discarded and the column was transferred to a fresh 2 ml collection tube. Afterwards, to completely dry the membrane, the QIAamp MinElute column was centrifuged in a new collection tube for 3 min at 16000 x g and then incubated at 56 °C for 3 min. Finally, the QIAamp MinElute column was transferred to a fresh RNase-DNase-free 1.5 ml Eppendorf tube (cat no:72.706.200; Sarstedt, Sarstedt, Germany) and 100 µl water (cat no:T143.4., Carl Roth, Karlsruhe, Germany) was added to the center of the membrane to elute viral RNA. After a 5 min incubation at ambient temperature, centrifugation was carried out at 16000 x g for 1 min. The obtained eluate was immediately frozen in equal parts on dry ice and stored at –80 °C for further analysis.

Detection and quantification of HEV RNA: Isolated RNA of all IgG- and IgM-reactive samples was then further analyzed with the IVD-certified RealStar HEV RT-PCR Kit 2.0 (cat no: 272013, Altona Diagnostics, Hamburg, Germany) for detection and if applicable the quantification of HEV-specific RNA according to the manufacturer's instructions with minor modification. Briefly,

12.5 µl of sample, Quantification Standard (QS) 1-4 or water (PCR grade) were mixed with 12.5 µl mastermix (consisting of internal control (IC), Master A, Master B) in a 96-well PCR plate (cat no: HSR9905, Bio-Rad, Hercules, USA). After sealing with PCR film (cat no: MSC-1001, Bio-Rad), the plate was centrifuged for 1 min (180 x g, 4 °C) in an Eppendorf Centrifuge 5804R. Samples were analyzed in duplicate, QS1-4 in triplicate wells.
Plates were run on a LightCycler 480 Instrument II (Roche, Basel, Switzerland) with the following setup:

- Reverse transcription: 55 °C for 20 min
- Denaturation: 95 °C for 2 min
- Amplification (45 cycles): denaturation (95 °C for 15 sec); annealing (55 °C for 45 sec); extension (72 °C for 15 sec)

C_p-values were calculated using the second derivative maximum method.

Supplementary Table 3. HEV IgG ELISA performance

**Results (n=technical replicates)**	**Axiom IgG ELISA**			**recomWell HEV IgG ELISA**		
	**Mean IgG S/CO**	**SD IgG S/CO**	**CV (%) IgG S/CO**	**Mean IgG S/CO**	**SD IgG S/CO**	**CV (%) IgG S/CO**
IgG^+ (n=4)	9.70	2.52	26.0	4.51	0.33	0.07
IgG^+ (n=4$^)$)	9.36	1.70	18.0	5.35	0.11	0.02
IgG^+ & IgM^+ (n=4)	14.54	2.03	14.0	8.55	0.75	0.09
Positive ctrl (n=18 Axiom; n=16 recomWell)	14.72	2.25	15.27	4.21	0.89	21.03
Negative ctrl (n=27 Axiom; n=16 recomWell)	0.01	0.01	87.95	0.04	0.01	17.96
HAVQC1 (17/B725) (n=9 Axiom; n=8 recomWell))	0.76	0.13	17.27	0.54	0.17	32.37
HEVQC1 (17/B723) (n=11 Axiom; n=8 recomWell)	4.52	0.73	16.10	2.68	0.85	31.73

References

1. EL CONGRESO DE COLOMBIA. LEY 142 DE 1994 [Internet]. Colombia; 1994. Available from: https://www.funcionpublica.gov.co/eva/gestornormativo/norma_pdf.php?i=2752.
2. Norder, H.*, et al.* Diagnostic Performance of Five Assays for Anti-Hepatitis e Virus IgG and IgM in a Large Cohort Study. *Journal of Clinical Microbiology* **54**, 549-555 (2016).
3. Mikrogen, D. Mikrogen - recomWell HEV IgG / IgM.
4. Team, R.C. R: A Language and Environment for Statistical Computing. (R Foundation for Statistical Computing, Vienna, Austria, 2021).
5. Lang, Z. & Reiczigel, J. Confidence limits for prevalence of disease adjusted for estimated sensitivity and specificity. *Preventive Veterinary Medicine* **113**, 13-22 (2014).
6. Fay, M.P. asht: Applied Statistical Hypothesis Tests. (2022).
7. Gwet, K.L. & PhD. Computing Chance-Corrected Agreement Coefficients (CAC). (2019).
